# Light People: Professor Liangcai Cao

**DOI:** 10.1038/s41377-023-01194-3

**Published:** 2023-06-05

**Authors:** Tingting Sun

**Affiliations:** Light Publishing Group, Changchun Institute of Optics, Fine Mechanics and Physics, Chinese Academy of Sciences, No. 3888 Dong Nanhu Road, Changchun, 130033 China

**Keywords:** Imaging and sensing, Displays

## Abstract

Holography utilizes the principles of wave interference and diffraction to record and reconstruct images, which can highly restore the three-dimensional features of objects and provide an immersive visual experience. Dennis Gabor proposed the concept of holography in 1947 and was awarded the Nobel Prize in Physics in 1971. Holography has gradually developed into two major research directions: digital holography (DH) and computer-generated holography (CGH). Holography has empowered the development of fields such as 6G communication, intelligent healthcare, and commercial MR headsets. In recent years, the general solution to optical inverse problems contained in holography also provides theoretical support for its wide integration with computational lithography, optical metamaterials, optical neural networks, orbital angular momentum (OAM), and other areas. This demonstrates its enormous potential for research and application. We are delighted to invite Professor Liangcai Cao from Tsinghua University, a leading scientist in the field of holography, to give us a profound interpretation of the opportunities and challenges of holography. In the interview, Prof. Cao will take us on a journey through the history of holography, share fascinating stories from his academic visits and exchanges, and shed light on the mentor and tutor culture in teaching. Through this episode of “Light People,” we will have the privilege of getting to know Prof. Cao on a deeper level.

## Abstract (web summary)

Prof. Liangcai Cao spoke about the current opportunities and challenges of holography and took us on a journey through the history of holography development.

## Graphical abstract

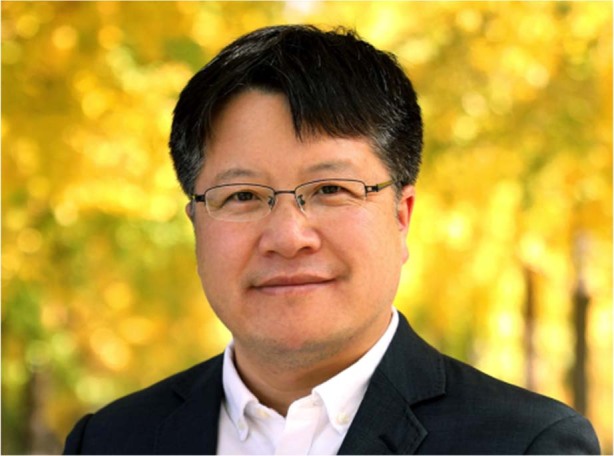

Liangcai Cao, professor of the Department of Precision Instruments, Tsinghua University, received his BS/MS and PhD degree from Harbin Institute of Technology and Tsinghua University, in 1999/2001 and 2005, respectively. Then he became an assistant professor at the Department of Precision Instruments at Tsinghua University. He is now a tenured professor and director of the Institute of Opto-electronic Engineering. He was a visiting scholar at UC Santa Cruz and MIT in 2009 and 2014, respectively. His research interests are holographic imaging and holographic display. He is a fellow of SPIE and OPTICA. Prof. Cao is the PI for a key project of the Natural Science Foundation of China and a Key Research and Development Project of the Ministry of Science and Technology. Prof. Cao has published over 100 journal papers and holds more than 40 patents. His achievements and contributions to the field have earned him numerous awards and recognitions, including the Champion Supervisor award by Light Publishing Group in 2021.

**Figure:**
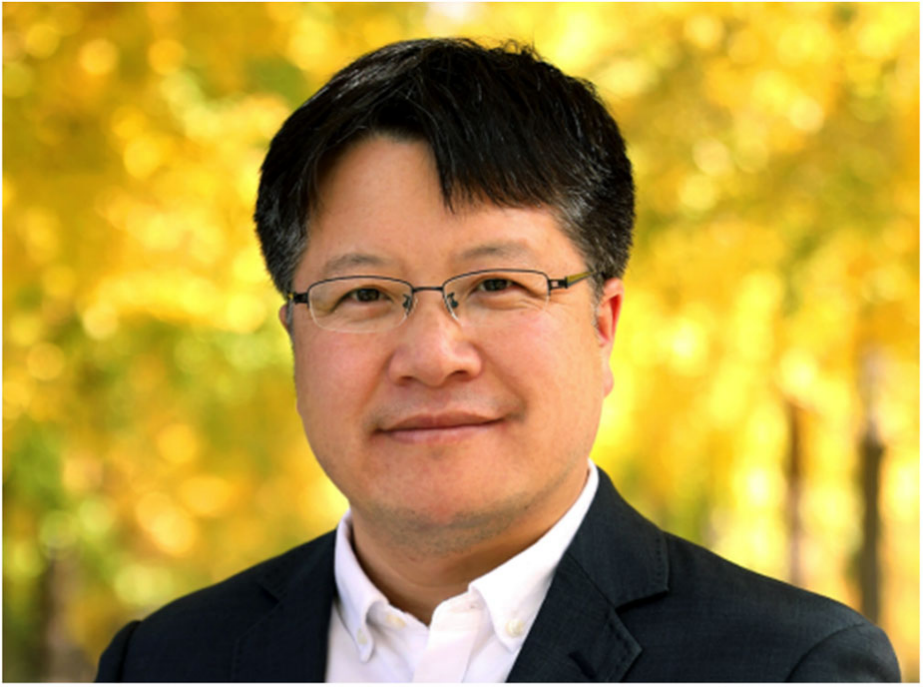

**Q1: Could you please briefly introduce your current research focus? What important progress has been made so far?**

**A1:** My research group, Hololab, is closely tied to optical holography. I began my Ph.D. studies in optical engineering at Tsinghua University in 2001 under the supervision of Prof. Guofan Jin and Prof. Qingsheng He. For my Ph.D. degree, I worked on volumetric holographic storage and optical computing. After graduation in 2005, I continued my research in these areas. In 2013, I started to participate in two joint projects led by Prof. Qionghai Dai and Prof. Yongtian Wang, respectively, and shifted my research focus to holographic imaging and holographic display. Over the past decade, Hololab’s main research interests have been focused on two technologies: computer-generated holography (CGH) for display and digital holography (DH) for imaging. The CGH display technology converts the digital model of an object into a two-dimensional hologram through computation, and projects the original three-dimensional object by using a spatial light modulator or a metasurface device. In contrast, DH imaging uses an image sensor to record the wavefront of the object as a 2D hologram, and then reconstructs the hologram through computation to obtain a digital model of the object. These processes involve novel algorithms and diffractive optical calculations. Our research is also applicable to general optical imaging and display technologies, which also fall under the field of information optics and holographic optics. We have successfully proposed holographic display algorithms and systems based on angular spectrum theory and deep learning, as well as developed a lensless imaging method based on compressive sensing and deep learning, which have been highly recognized by peers. Currently, we have strong connections with the industry.

**Figure:**
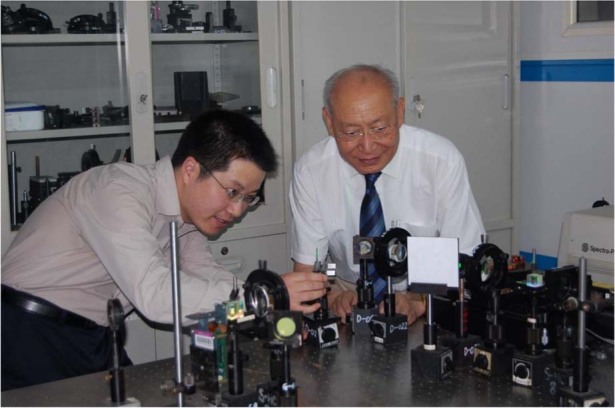
Prof. Guofan Jin and Liangcai Cao were working on holographic data storage at Tsinghua University (September 2008)

**Q2: Coded aperture imaging, as a lensless imaging technology, is characterized by its compact optical setup and easy implementation. How to establish the connection between the scene and the image, and how to reconstruct the image by solving the inverse problem is the key issue in encoded imaging. You and Prof. George Barbastathis jointly proposed an encoded imaging technology using a Fresnel zone plate (FZP), which realized lensless imaging under incoherent illumination**^1^**, and had it published on** ***Light: Science & Applications (Light for short)*. Can you explain the main idea of this research? What are the advantages of this technology?**

**A2:** Coded aperture imaging is a technique that replaces traditional optical lenses with a mask containing a specific pattern in front of the sensor The mask modulates the incident light, forming a pattern on the image sensor. A reconstruction algorithm is used to recover the original image from the coded image. The challenge for the coded aperture imaging is how to model the pattern from the scene and reconstruct the scene by solving the inverse problem. Prof. David Brady and our group developed an algorithm based on compressive sensing to realize twin-image free digital holography^2^, which was published in *Physics Review Letters* in 2018. Following this, we sought to determine whether this approach could be applied to lensless imaging. Our studies revealed that the ill-posed inverse problem of lensless imaging can be successfully solved using TV regularization. This achievement is due to the disparity in sparsity between the twin image and the original image, particularly in the gradient domain.

In the research published in *Light*, we employed a single Fresnel zone plate (FZP) to achieve lensless imaging under incoherent illumination. The imaging system consisted of only an image sensor and an FZP placed a few millimeters in front of the sensor. By exploiting the similarity between the point source hologram and the FZP structure, we used the FZP as a coding mask to encode the object. The coded image has the same form as the in-line hologram. Using compressive sensing, the algorithm eliminates the twin-image artifact in holographic reconstruction and achieves high-quality image reconstruction with minimal background noises. The main advantage of this camera is that it does not require calibration and can obtain an image with a single exposure. In the future, it can be easily integrated into mobile devices, monitoring cameras, autonomous vehicles, and other applications.

**Q3: Digital holographic imaging uses computational methods to reconstruct the amplitude and phase of the object wave, which can quantitatively analyze the interaction between light and object. Can you address the main application fields of digital holographic imaging technology? What important impacts has brought to our daily life by its development?**

**A3:** The phase of a light wave mainly carries information about the optical path difference, which is related to changes in the refractive index or in the thickness of an object. In the field of biomedical imaging, there is a certain difference between the refractive index of different biological tissues and the external environment. Digital holographic imaging technology can use this difference to realize label-free observation of various biological phenomena. Compared with chemical staining, fluorescent staining, and other observation methods, digital holographic imaging technology creates minimal disruption on the normal physiological activities of cells. In optical detection and metrology, digital holography has proven to be an effective tool for precise surface measurements at the nanometer scale, as thickness fluctuations in a sample can be reflected in phase changes. In addition, since digital holography records the entire light field, it can be used for light field inversion to achieve 3D imaging, which is an active research area in academia.

The DH technology has gained popularity in various fields due to its high resolution and ability to provide phase information that traditional imaging methods cannot obtain. It has found applications in interferometry, microparticle detection, and biomedical imaging. Digital holographic microscopy has also made its way from the laboratory to industry, and DH’s unique superiority in data processing enables its combination with other technologies to support precision metrology, microstructure imaging detection, and medical diagnosis.

**Q4: As a leading scientist in the field of digital holographic imaging, can you comment on the progress of this technology? In your opinion, what is the future trend of development?**

**A4:** Holographic imaging has been developed for more than 70 years since its inception, and the corresponding imaging technology has made significant advances over the years. However, due to the limitations of complex optical systems and bulky optical components, most holographic imaging technologies are still primarily limited to laboratory settings. In recent years, digital holographic imaging has regained considerable attention following the development of computational imaging. For instance, the Digital Holography and 3D Imaging Conference, one of the topical meetings by OPTICA, is held annually worldwide, where researchers from the academic and industrial communities worldwide come together to exchange ideas.

As digital holographic imaging technology advances, many companies have started to explore industrial applications in biomedical imaging, optical detection, and metrology. Companies such as Nanolive in Switzerland and Tomocube in South Korea, as well as Chinese companies such as BayJayRay, Institute of Intelligent Imaging of Nanjing University of Science and Technology, and many other enterprises have successively launched a series of digital holographic microscopic imaging instruments. To facilitate the industrial implementation of digital holographic imaging, it is crucial to collaborate with users from various application fields. A key challenge in promoting DH technology to biomedical laboratories is establishing a specificity between optical phase and the target that biologists or doctors are interested in.

In the future, we also hope to combine holographic imaging technology with nanofabrication techniques. This will make the systems more compact and fully leverage the advantages of holographic imaging, such as large field of view and high resolution.

**Figure:**
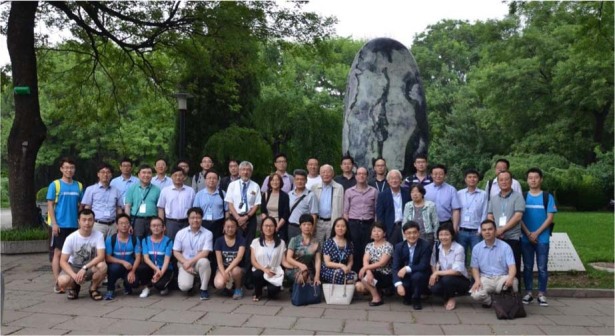
Researchers of holography met at Tsinghua University (July 2018)

**Q5: In recent years, computational imaging technology has developed rapidly. Can you provide a brief introduction on the past and present of computational imaging technology? As is presented in your recent paper published at Light: Advanced Manufacturing, the iterative projection and sparsity regularization are useful tools for practical single-shot quantitative phase imaging with in-line holography** ^3^ **, which caused broad attentions. The combination of compressive sensing and deep learning has offered new ideas for research in computational imaging technology. What are the specific manifestations?**

**A5:** The development of computational imaging is tightly interlinked with the advancements in semiconductor and Internet technologies. On the one hand, the steady progress in submicron, deep submicron, and nanotechnology processes, as well as the improvement of device structures, the image sensor technology has become more and more mature. Parameters such as resolution, signal-to-noise ratio, and dynamic range still keep improving constantly, while other dimensions such as phase, polarization, and spectrum can now be captured by light intensity and recorded by the image sensor. This enables us to acquire information about the light field in the real world with the help of specific optical components or additional imaging devices. On the other hand, with the rise of mobile network and the improvement of computer processing power, research fields such as image processing, computer vision, machine learning, and big data processing have made significant advances. The mutual collision and integration of these two research fields have driven the birth and development of computational imaging.

Compressive sensing and deep learning, as typical representatives of model-driven and data-driven methods, are widely used in computational imaging. As theoretically verified by compressed sensing and practically demonstrated by deep learning, prior knowledge of signals can be utilized to retrieve complex signals with undersampled measurements. As early as 2009, Prof. David Brady proved theoretically that the encoding method of holographic diffraction satisfies the conditions of compressive sensing, which led to the proposal of “compressive holography”. Subsequently, corresponding applications such as single-pixel imaging, spectral imaging, and depth imaging emerged. However, compressive sensing also has its shortcomings, such as its dependence on prior knowledge and long computation time, which have hindered its development. Deep learning can learn the latent mapping relationships contained in a large dataset through training. As a data-driven approach, deep learning gets rid of the dependence of analytical and numerical solution methods on forward models and prior information, providing a new framework for solving inverse problems. Deep learning has achieved superior performance in some imaging applications with complex models, such as imaging through scattering media and imaging at low photon count. However, in recent years, the uninterpretability of deep learning has gradually attracted attentions, and how to organically combine imaging models and the superior performance of deep learning is the direction for future research.

**Figure:**
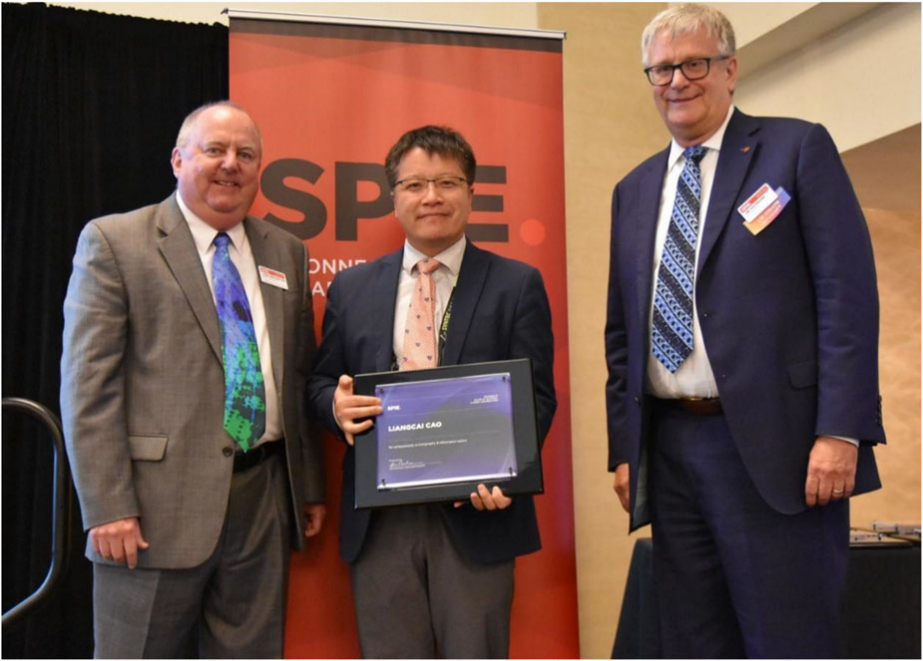
Prof. Liangcai Cao was awarded the SPIE Fellow (August 2019)

**Figure:**
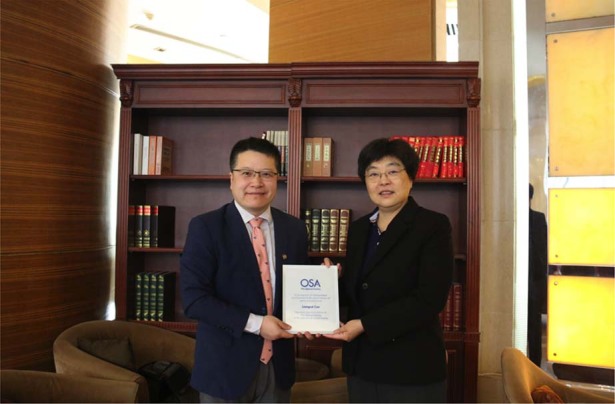
Prof. Liangcai Cao was awarded the OSA (Optica) Fellow (October 2020)

**Q6: Your team has successfully implemented deep learning technology to achieve fast and high-quality 3D imaging and display, what role can deep learning technology play? What opportunities and challenges does the holographic dynamic 3D display technology face?**

**A6:** An ideal human-computer interaction device should be able to promptly respond to user instructions and switch 3D display images in a fast and high-quality manner. This process not only involves imaging and display, but also demands high accuracy and timeliness of technologies such as modeling, machine vision, motion capture, and semantic understanding. Deep learning technology provides innovative solutions to these challenges. For example, 3D imaging effects are often limited by equipment and environments, resulting in issues such as color distortion, uneven brightness, and depth confusion. Overcoming these problems requires substantial computing power, and it is difficult to meet the demands of immersive interaction in terms of speed. Deep learning-based imaging enhancement technology can automatically filter out and repair abnormal images, improving the speed and quality of 3D imaging, which provides critical support for immersive human-computer interaction.

Compared with 2D displays, 3D displays can provide image content that is closer to the real world and are the important support for establishing an immersive and realistic experience in the metaverse. Holographic 3D display is capable of providing all kinds of depth cues, resulting in a more user-friendly and realistic visual experience, making it one of the ideal solutions for 3D display. Compared with the current commercial head-mounted display solutions based on binocular vision or light field display principles, CGH avoids the conflict of convergence adjustment in principle, allowing viewers to obtain an immersive interactive experience without dizziness and other visual fatigue. Challenges currently for achieving high-quality holographic dynamic 3D displays include limited holographic reconstruction quality, modulation performance of wavefront modulation devices, spatial bandwidth of holographic display systems, and holographic 3D content sources. Despite these challenges and limitations, recent breakthroughs suggest that holographic 3D display can gradually approach its ideal form, and it is expected to become a fundamental technology in the field of metaverse, with broad prospects for application in intelligent manufacturing, remote education, remote work, entertainment, and social interaction.

**Q7: Your team proposed a new type of lensless fiber optic micro-endoscope imaging technology** ^4^ **, the endoscope is as small as an embroidery needle but has an ultra-high magnification of 1000 times. This allows doctors to view the cells on the surface of the tissue with great precision and reduces patient discomfort during the medical procedures. Can you describe the innovation of this work? What level of accuracy is currently achievable? For the follow-up research, what key issues remain to be resolved?**

**A7:** This work combines inline holography, phase retrieval algorithms, and fiber bundle imaging to achieve quantitative phase imaging (QPI) based on fiber optic endoscopes. QPI can provide rich image contrast information, such as cell dynamic contrast, reflectance contrast, refractive index contrast, phase information, etc. It allows cancer cells to be distinguished from a complex background. In addition, important physical parameters such as cell volume, refractive index, and mass can also be calculated from quantitative phase reconstruction images, which can provide more valuable auxiliary information for clinical diagnosis and research. One of the significant advantages of this technology is its ability to increase the working distance of this lensless fiber-optic micro-endoscope significantly. The working distance has been significantly increased from within 10 microns to 10 mm. Objects as small as one micron can be “seen”, and nanoscale three-dimensional reconstruction has been achieved. In subsequent research, we will further study the light transmission characteristics of the optical fiber and improve the lighting method to better apply this technology to the in-situ observation.

**Q8: We know that you have been a visiting scholar at UCSC and MIT, can you share this story with us? What was the most significant thing you learned during your visiting there? Compared with domestic research, what are the characteristics of the foreign scientific research environment?**

**A8:** I visited Prof. Claire Gu’s research group at UC Santa Cruz (2009) and Prof. George Barbastathis’ research group at MIT (2014), which was very helpful for my international academic exchanges. I got to know more and more international scholars and learn from them. The two visits offered me chances to get to know new research topics. I also kept in touch with foreign tutors even after I came back to China. They continue to give us instructions, and we have effective cooperation.

During my visit to the United States, I begin to realize that research itself is a relatively pure, simple, and enjoyable career. The faculty and students in the research group paid attention to scientific problems and worked on key issues in the field. Although there were not too many people in the research groups I visited, they were all very interested in academia and could carry out long-term, in-depth and high-impact research on a specific topic. My colleagues were easygoing, and everyone called each other by their first names. Teachers and students were active and highly concentrated in their work, which showed their professionalism. Therefore, I strongly encourage the students at Hololab to go abroad for visits and studies. This can cultivate their global perspective and is beneficial to their future career developments.

**Figure:**
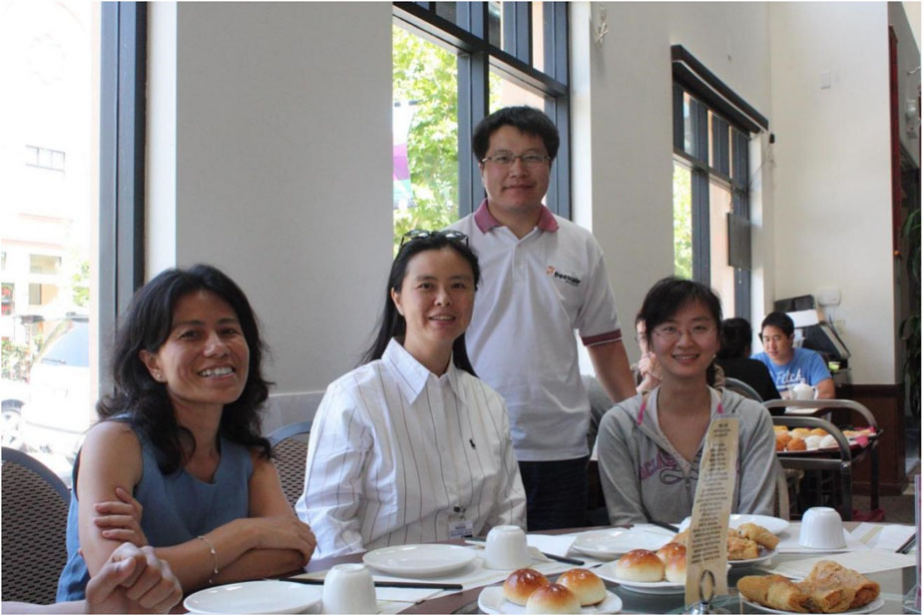
Prof. Liangcai Cao and Prof. Claire Gu at UC Santa Cruz (August 2019)

**Figure:**
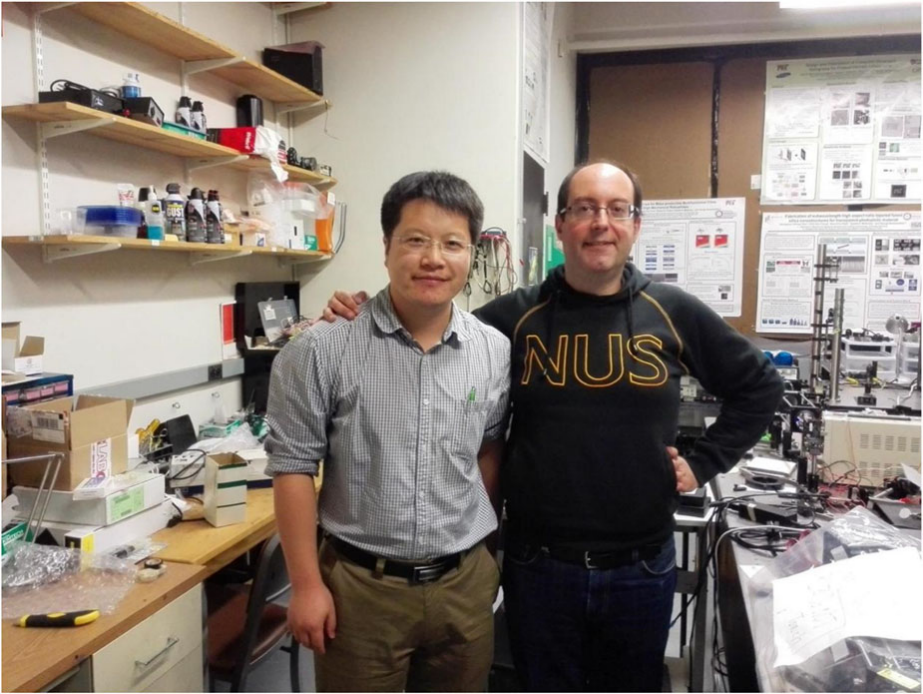
Prof. Liangcai Cao and Prof. George Barbastathis at MIT (September 2014)

**Q9: In the 2021 Light Doctoral Academic League held by Light, the Beijing Division you led achieved excellent results, and you also won the Award of Champion Mentor. In your opinion, what is the positive significance of holding such academic activities?**

**A9:** The Academic League provides a platform for outstanding PhD students engaged in optics and optical engineering to showcase their academic abilities and exchange ideas. Students take advantages of this platform to fully express their research motivation, innovations and conclusions for their doctoral theses. This is a very rare opportunity to cultivate excellent doctoral students. The role of the academic league is to provide an opportunity for academic reports. Tens of thousands of viewers watched these academic presentations online. There were questions, suggestions, and comments from the scholars and professors, as well as competitions with outstanding students. Therefore, these academic presentations surpassed other presentations to a certain extent. It must be of great help to improve the level of students’ doctoral theses and academic presentations. China is currently cultivating the next generation of innovative optical talents. In addition to being diligent in thinking and daring to practice, they must be good at expressing themselves, sharing their own ideas. Thus, every candidate should actively participate and think about how to present their own research, which will have a positive impact on academic peers and even the whole society. In addition, the innovative form of competition in ten sections in China also promoted communication and understanding among the participants, forming a good relationship between competition and cooperation. This event allows doctoral students not only to earn honors, but also to meet like-minded peers.

**Figure:**
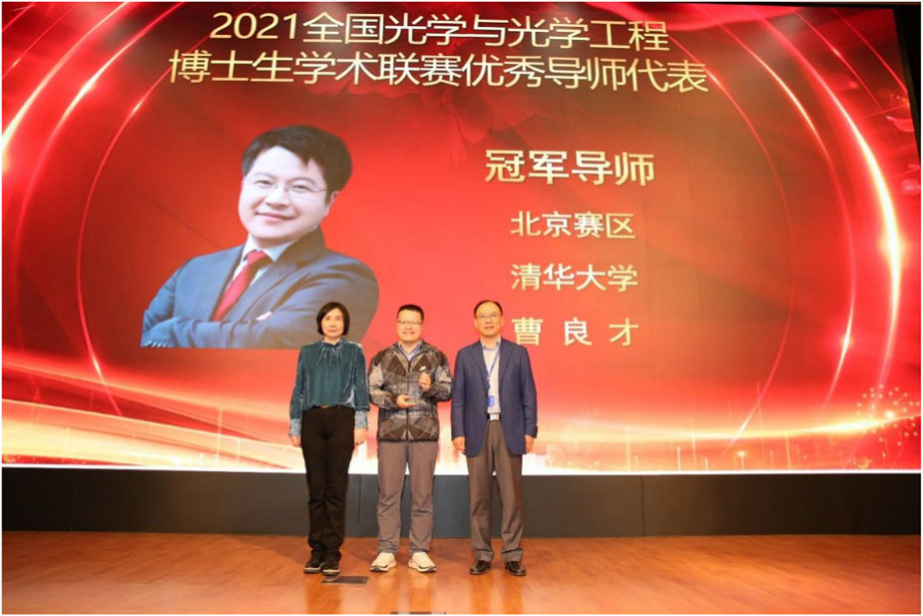
Prof. Liangcai Cao won the 2021 Academic League Championship Mentor (May 2021)

**Q10: Innovative talents have two crucial characteristics: one is the ability to “create”, which means surpassing himself, and the other is to be “novel”, which means being distinguished. Regarding the mentoring, you suggest that “teachers and students are mentors to each other for innovation.” Could you explain this distinctive philosophy to us?**

**A10:** I strongly encourage undergraduates to participate in extracurricular creative and research activities. Through the exchange and interaction of scientific research activities, they can constantly shape the correct value orientation and personality traits such as courage, perseverance, self-confidence, and teamwork. During this process, they can solidify their fundamental knowledge and resolve to learn more, so as to have life-long learning ability and the innovative potential of sustainable development. Students at Tsinghua University choose their own majors after passing the college entrance examination. They may not have a deep understanding of the majors they study. Therefore, I would prefer to help them identify their research interests and improve their scientific research abilities. If they have less interest in the major, no matter how hard they work and how smart they are, they can hardly obtain much sense of achievements from scientific research. The urge for scientific research often comes from discovering the imperfections of the world and being eager to change it for the better. The motivations of a large number of top scholars at Tsinghua University determine what the university is like nowadays. In addition, the career development directions of some students are a bit ambiguous. I always encourage students to clearly set their ambitions, go for the big stage, and succeed in their careers. According to the academic masters and typical cases in this field, I often use the saying “chase the light for the whole life and the whole world” to encourage students to push forward the advancement of optics and use “light” to lighten their lives. It is amazing to pursue a shining career life in optics. When choosing a career, the students should not only consider the present benefits, but “think about the world” and strive to make a difference.

**Figure:**
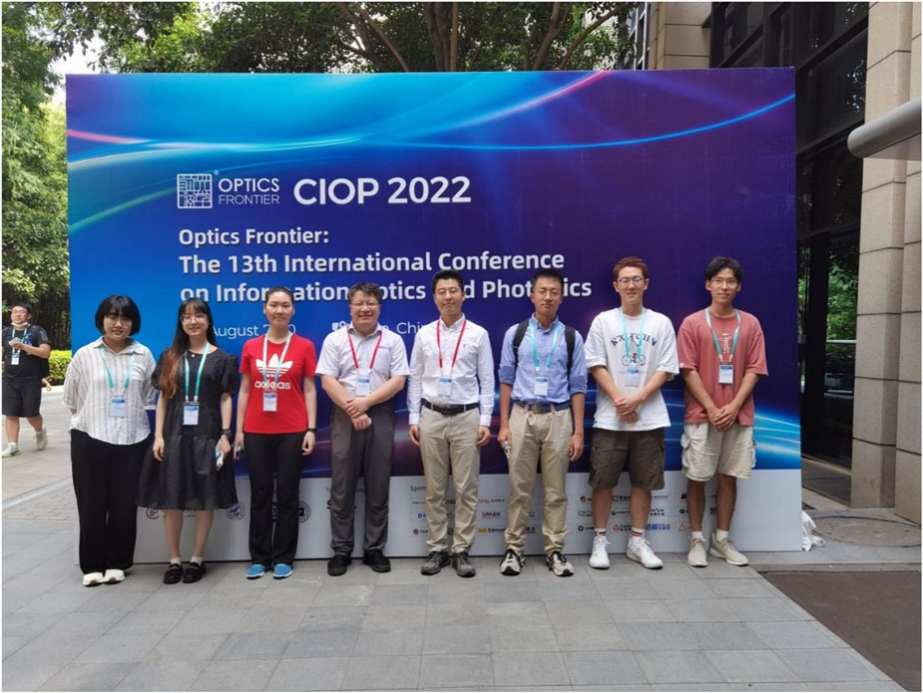
Prof. Cao, postgraduate students, and undergraduate students participated in the CIOP2022 conference in Xi’an (August 2022)

**Q11: In July 2022, Light Science Forum was founded to provide clear explanations, credible answers, and reliable reviews for common problems in our daily lives. We know that making science accessible to the public is a tall order. As a member of the Scientist Advisory Board, what suggestions do you have for this?**

**A11:** Popularizing science is a fundamental responsibility of every researcher. Only when one’s work is clearly explained to the public, can this work possess a sufficiently high social value. I encourage PhD students at Hololab to simplify and introduce their works to friends and families. Our research findings can only be widely disseminated when they are understood by the people around us. Teaching and learning are mutual. The amount of knowledge taught by the teacher does not imply the amount of knowledge learned by the students. However, the latter is what really matters. This is also applicable to scientific research. The quantity of scientific research findings does not equal to the application of those findings. We are often asked to explain complicated works in one or two sentences in a conference or a seminar. The greatest truth is concise. On the other hand, science popularization not only serves to stimulate public interest in technology, but also encourages wider participation in scientific innovation. This can finally bring about more investment in scientific research and push forward the development of technology. There is still an increasing amount of social capital, individuals, and groups supporting the public scientific and technological research through donations, which is also a very long-term return for science popularization. I have gained a lot of insights when I visited the Optics Museum at Changchun. I hope that the Light Science Forum can collect and provide more updated scientific knowledge. At the same time, we may use International Light Day to build a bridge with the world’s optical science popularization. We will work together to let more people see light, know light, and chase light.

**Q12: Thank you for your support and contribution to** ***Light. Light*** **has gone through 11 years from its ignorant appearance to its international reputation, we would like to hear your thoughts and expectations for the journal. How do you envision** ***Light*’s future, and what impact do you hope it will have in the field of optics?**

**A12:** The launch of Light is a major milestone in the history of Chinese optics. We are excited to see that Light makes its mark on the world stage by demonstrating China’s wisdom, efforts, and contributions in global optical development. As a global community, Light constantly makes the world better by the research and utilization of optics and photonics.

**Figure:**
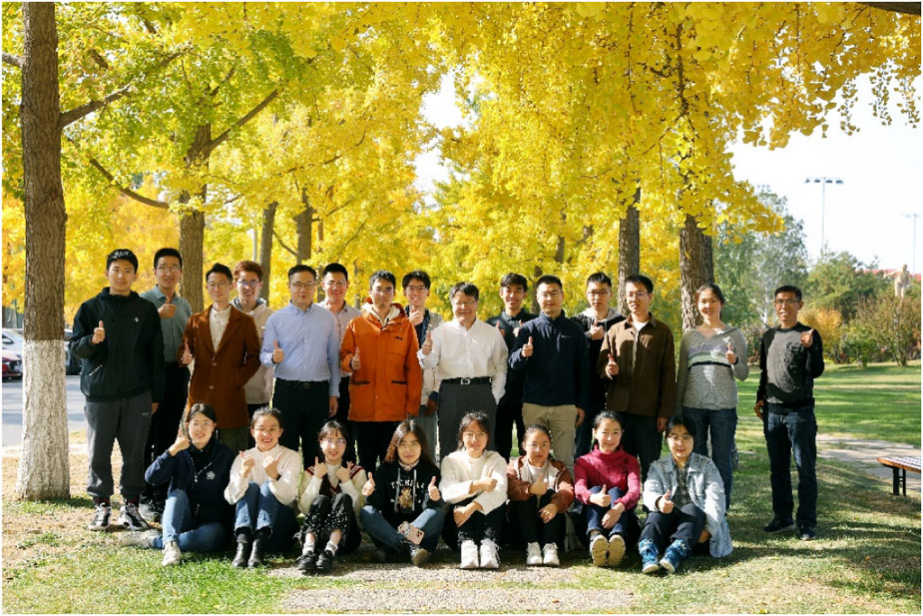
Group photo of Hololab (October 2022)
